# Patient Satisfaction With Rhinoplasty and Influencing Factors; Using the Rhinoplasty Outcome Evaluation Questionnaire: A Cross‐Sectional Study in Iran

**DOI:** 10.1002/hsr2.71517

**Published:** 2025-11-17

**Authors:** Milad Rezai, Afrooz Haghdoost, Mohammad Tolouei, Enayatollah Homaie Rad, Ramyar Farzan, Maria Ahmadi Jirandeh

**Affiliations:** ^1^ School of Medicine Guilan University of Medical Sciences Rasht Iran; ^2^ Department of General Surgery, School of Medicine Guilan University of Medical Sciences Rasht Iran; ^3^ Department of Plastic & Reconstructive Surgery, School of Medicine Guilan University of Medical Sciences Rasht Iran; ^4^ Social Determinants of Health Research Center, Trauma Institute Guilan University of Medical Sciences Rasht Iran; ^5^ Burn and Regenerative Medicine Research center Guilan University of Medical Sciences Rasht Iran

**Keywords:** Iran, outcome, patient satisfaction, questionnaire, rhinoplasty

## Abstract

**Background and Aims:**

The present study aimed to investigate patients' satisfaction after rhinoplasty using the ROE questionnaire and to examine some influencing variables.

**Methods:**

This is an analytical cross‐sectional study. The study population included all patients seeking rhinoplasty referred to the plastic surgery department of Velayat Hospital in Rasht in 2023. Initially, the demographic characteristics of the patients were recorded. The Rhinoplasty Outcome Evaluation questionnaire was used to assess patient satisfaction. All patients were operated on by a single experienced plastic and reconstructive surgeon using the open rhinoplasty technique. The ROE questionnaire was completed by all patients before surgery and then 3 months after surgery during a follow‐up visit. Descriptive and analytic statistics were used for data analysis, with a significance level of *p* < 0.05. Statistical analyses were performed using the IBM SPSS Statistics software, version 28.

**Results:**

One hundred and thirty patients were examined. Eighty‐five of the participants (65.4%) were female. The mean age of the patients undergoing rhinoplasty was 26.5 ± 6.24 years. The obtained score before rhinoplasty in patients was 32.76 ± 12.63, and 3 months after surgery, it was 92.05 ± 5.83, which was statistically significant (*p* < 0.001). Significant differences were found in patient satisfaction scores before and 3 months after surgery in each group for the variables under study (gender, age, marital status, and education). Additionally, in the between‐group comparison in male and female patients, the mean percentage increase in patient satisfaction was 68.14 ± 9.73 in males and 54.6 ± 6.15 in females, which was statistically significant (*p* < 0.001).

**Conclusion:**

This study showed that despite the complexity and intricacies of rhinoplasty, it leads to high satisfaction levels for most patients, and some demographic factors significantly affect patient satisfaction with rhinoplasty outcomes. However, generally, rhinoplasty has a positive effect on patient satisfaction regardless of age, gender, and education level.

## 1 Introduction

Rhinoplasty is one of the most commonly performed surgical procedures worldwide, used to correct nasal deformities for either aesthetic enhancement or functional improvement. It is considered one of the most delicate and complex cosmetic surgeries due to the three‐dimensional structure of the nose, the need for precision, and the variation in individual anatomy [1, 2]. Iran is among the leading countries in cosmetic surgery, particularly rhinoplasty. Although official statistics are lacking, multiple studies report a sharp increase in rhinoplasty procedures in Iran over the past decade, with some data suggesting it accounts for more than 60% of all cosmetic surgeries and placing the country among the top three globally for such procedures [3, 4, 5].

Although evaluations of rhinoplasty outcomes focused primarily on surgical techniques, clinical protocols, complication rates and the necessity for revision surgeries, nowadays the assessment of patients' satisfaction and their opinion regarding the final outcome has gained increasing significance. After surgery, patients may express dissatisfaction with nasal appearance, airway functionality, nostril asymmetry or scarring [1]. Patient dissatisfaction poses a significant challenge to surgeons and physicians and nowadays due to the increasing popularity of rhinoplasty, patients' complaints are reported more than before [1, 6]. Consequently, evaluating surgical outcomes assumes paramount importance in plastic surgery, with patient satisfaction emerging as an important criteria for success or failure of the procedure [7].

In recent decades, the focus has shifted towards assessing patient satisfaction with surgical outcomes, prompting plastic surgeons to accord greater attention to this facet. Divergent views between surgeons and patients on defining a successful outcome underscore the necessity for standardized evaluation tools. In this regard, Alsarraf and colleagues developed a tool called the Rhinoplasty Outcome Evaluation (ROE), which has demonstrated high reliability, internal consistency, and validity in assessing the outcomes of this surgery. It has been introduced as a standard, reliable, and quick‐to‐use tool for evaluating patient satisfaction after rhinoplasty [8].

Numerous studies have been conducted all over the world about rhinoplasty outcomes. But in spite of the importance of cosmetic surgery in Iran, few studies have investigated the patients' satisfaction and influencing variables after cosmetic surgeries, using standard questionnaires in this country. Therefore, this study aims to investigate patient' satisfaction utilizing the ROE questionnaire and explore influencing variables among patients referred to the plastic surgery department of Velayat Hospital in 2023. The findings of this study offer insights into the prevailing satisfaction levels among rhinoplasty patients in our region and enable comparative analyses with international standards.

Methods: The present study adopts an analytical cross‐sectional design. The statistical population included all patients seeking rhinoplasty who visited the plastic surgery department of Velayat Hospital in Rasht during 2023.

Inclusion criteria were applicants of both genders, aged 18–60 years, willing to participate. Exclusion criteria included individuals under 18 or over 60 years old, those with congenital facial abnormalities, candidates for secondary rhinoplasty, and individuals with a history of severe mental illness or addiction. The study was approved by the Ethics Committee of the Research and Technology Department at Guilan University of Medical Sciences (Approval Number: IR.GUMS.REC.1401.574; Date: February 15, 2023), and informed consent was obtained from all participants.

Demographic characteristics were initially recorded. The Rhinoplasty Outcome Evaluation (ROE) questionnaire was used to assess patient satisfaction. All patients underwent open surgery performed by a single experienced plastic and reconstructive surgeon. The questionnaire was completed once before surgery and again 3 months postoperatively during an in‐person visit.

The ROE is a standard, reliable, and easy‐to‐use tool for assessing satisfaction with rhinoplasty outcomes. It consists of six questions rated on a 5‐point Likert scale, evaluating satisfaction across physical, psychological, and social criteria—two questions each. Scores (0–4) are summed (total 0–24), divided by 24, and multiplied by 100 to yield a final score (0%–100%). Lower scores indicate less satisfaction; higher scores, greater satisfaction [8, 9, 10].

The ROE questionnaire has been translated into multiple languages, with studies confirming its excellent psychometric properties. Reported test‐retest reliability ranges from 0.74 to 0.83 (Pearson correlation coefficient), and internal consistency scores range from 0.83 to 0.88 (Cronbach's *α*). Responsiveness to change has also been statistically significant (*p* < 0.001), with patient satisfaction increasing from an average of 37% to over 84% after surgery. The validity and reliability of the Persian version have also been confirmed, with a Cronbach's *α* of 0.727 and an average CVI of 0.8 [9, 10].

Statistical analysis included both descriptive and analytical methods. Descriptive statistics summarized demographic variables using measures of central tendency and dispersion. The Kolmogorov‐Smirnov test assessed normality. Based on the study design, *χ* ^2^, Kruskal–Wallis, and Fisher's Exact tests were applied. All analyses were performed using IBM SPSS Statistics, version 28.

Result: In this study, 130 patients were examined, of whom 85 (65.4%) were women. The average age of the patients was 24.6 ± 5.02 years, with the youngest being 17 years old and the oldest 50 years old. Among the participants, 82 (63.1%) cited cosmetic issues as the only reason for their visit, while 41 (31.5%) mentioned cosmetic alongside breathing issues, and 7 (5.4%) cited breathing problems alone as the motive for their visit. Patient characteristics are summarized in Table 1.

**Table 1:** Frequency distribution of the characteristics of rhinoplasty candidate patients.

Variable	Status	*N*	Percent
Gender	Male	45	34.6
	Female	85	65.4
Age (years)	less than or equal to 25	89	68.5
	More than 25	41	31.5
Age (years) Mean ± SD (min–max)		24.6 ± 5.02 (17–50)	
Marital status	Single	111	85.4
	Married	19	14.6
Education level	up to diploma	35	26.9
	Bachelor's degree	92	70.8
	Masters	3	2.3
The reason for requesting rhinoplasty	Cosmetic rhinoplasty	82	63.1
	Breathing problem	7	5.4
	Cosmetic and breathing problem	41	31.5

Table 2 presents the frequency distribution of the reasons for seeking surgery among patients across different variables. The data indicate a statistically significant association between the reason for rhinoplasty and different age groups (*p* < 0.0001). Specifically, patients opting for rhinoplasty for cosmetic purposes had a mean age of 23.65 ± 4 years, whereas those seeking it for breathing issues had a mean age of 33.71 ± 12.25 years. Moreover, a statistically significant difference in the reasons for seeking rhinoplasty was observed between single and married patients (*p* = 0.033). However, no significant relationship was found between patients' gender and the reason for rhinoplasty (*p* = 0.289), nor between their educational level and the reason for seeking surgery (*p* = 0.611).

**Table 2:** Frequency distribution of reasons for requesting rhinoplasty in patients based on gender, age and education.

Variable	Reason for rhinoplasty status	Cosmetic		Breathing problem		Cosmetic and respiratory problem		*p*‐value
		*n*	%	*n*	%	*N*	%	
Gender	Male	25	58.1	2	4.4	18	40	*p* = 0.289
	Female	57	67.1	6	5.9	23	27.1	
Age (years)	less than or equal to 25	65	73	3	3.4	21	23.6	*p* = 0.002
	More than 25	17	41.5	4	9.8	20	48.8	
Age (years) Mean ± SD (min–max)		23.65 ± 4.0		33.71 ± 12.25		24.92 ± 3.14		*p* < 0.0001
Marital status	Single	74	66.7	4	3.6	33	29.7	*p* = 0.033
	Married	8	42.1	3	15.8	8	42.1	
Education level	Up to diploma	24	68.6	2	5.7	9	25.7	*p* = 0.611
	Bachelor's degree	57	62	5	5.4	30	32.6	
	Masters	1	33.3	0	0	2	66.7	

Tables 3 and 4 depict the frequency distribution of responses from rhinoplasty candidates and the average scores and percentages derived from the ROE questionnaire before and 3 months after surgery. According to Table 4, there was a statistically significant difference in the average percentage scores obtained from the ROE questionnaire before and 3 months after rhinoplasty surgery (*p* < 0.0001). The data shows that the average percentage score increased from 32.76 ± 12.63 percent before surgery to 92.05 ± 5.83 percent 3 months post‐surgery.

**Table 3:** Frequency distribution of patient responses to the ROE questionnaire before and three months after rhinoplasty.

Questions 1 to 6	Time	Scores									
		0		1		2		3		4	
		*n*	%	*n*	%	*N*	%	*n*	%	*n*	%
1. How well do you like the appearance of your nose?	Before rhinoplasty	58	44.6	32	24.6	40	30.8	0	0	0	0
	After 3 months	0	0	0	0	0	0	76	58.5	54	41.5
2. How well are you able to breathe through your nose?	Before rhinoplasty	5	3.8	26	20	57	43.8	22	16.9	20	15.4
	After 3 months	0	0	0	0	3	2.3	21	16.2	106	81.5
3. How much do you feel your friends and loved one like your nose?	Before rhinoplasty	30	23.1	40	30.8	60	46.2	0	0	0	0
	After 3 months	0	0	2	1.5	0	0	56	43.1	72	55.4
4. Do you think your current nasal appearance limits your social or professional activities?	Before rhinoplasty	1	0.8	23	17.7	31	23.8	35	26.9	40	30.8
	After 3 months	0	0	3	2.3	2	1.5	2	1.5	123	94.6
5. How confident are you that your nasal appearance is the best that it can be?	Before rhinoplasty	87	66.9	16	12.3	27	20.8	0	0	0	0
	After 3 months	0	0	0	0	3	2.3	0	0	0	0
6. Would you like to surgically alter the appearance or function of your nose?	Before rhinoplasty	89	68.5	38	29.2	3	2.3	0	0	0	0
	After 3 months	0	0	0	0	0	0	11	8.5	119	91.5

**Table 4:** The average scores obtained from the ROE questionnaire by patients before and three months after rhinoplasty and their comparison.

Time	Number of questions	Total	The average total score of the patients (and the average percentage) Mean ± SD	Minimum score	Maximum score	*T* test	*p*‐value
Before rhinoplasty	6	24 (100%)	7.86 ± 3.03 (32.76 ± 12.63)	2 (8.33)	14 (58.33)	*t *= 43.7	*p* < 0.0001
Three months after rhinoplasty	6	24 (100%)	22.09 ± 1.4 (92.05 ± 5.83)	17 (70.83)	24 (100)		

Table 5 displays the percentage scores obtained from the ROE questionnaire among different groups of rhinoplasty patients, along with comparisons of the obtained scores. The average score of male patients before surgery was 26.57 ± 8.62 and 3 months after surgery was 94.72 ± 3.37. In female patients, the percentage of scores obtained from the ROE questionnaire before surgery and 3 months after surgery was 36.02 ± 13.21 and 90.63 ± 6.36, respectively. Notably, significant differences were observed in the average scores before and after surgery among male patients (*p* < 0.0001) and female patients (*p* < 0.0001). Additionally, the average percentage increase in the ROE questionnaire score was 68.14 ± 9.73 in men and 54.6 ± 15.9 in women. So between‐groups study revealed significant differences in the average percentage increase in ROE questionnaire scores between male and female patients (*p* < 0.0001).

**Table 5:** Comparison of the percentage of scores obtained in the ROE questionnaire by patients before and 3 months after rhinoplasty in different gender, age and education groups.

Variables	Group	*n*	The score obtained from the ROE		*p*‐value within‐Group	The rate of increase of scores (%)	*p*‐value between group
			Before rhinoplasty Mean (%) ± SD	After 3 months Mean (%) ± SD			
Gender	Male	45	26.57 ± 8.62	94.72 ± 3.37	*t* = 46.9 *p* < 0.0001	68.14 ± 9.73	*t* = 5.21 *p* < 0.0001
	Female	85	36.02 ± 13.21	90.63 ± 6.36	*t* = 31.65 *p* < 0.0001	54.6 ± 15.9	
Age	less than or equal to 25	89	31.46 ± 11.73	92.36 ± 5.93	*t* = 39.2 *p* < 0.0001	60.9 ± 14.64	*t* = 1.76 *p* = 0.079
	More than 25	41	35.56 ± 14.13	91.36 ± 5.62	*t* = 21.33 *p* < 0.0001	55.79 ± 16.74	
Marital Status	Single	111	33.14 ± 12.13	94.77 ± 5.93	*t *= 41.15 *p* < 0.0001	58.63 ± 15	*t* = 1.18 *p* = 0.24
	Married	19	30.48 ± 15.4	93.64 ± 5.07	*t* = 15.46 *p* < 0.0001	63.15 ± 17.8	
Education	up to diploma	35	30.11 ± 13.85	90.59 ± 5.92	*t *= 20.6 *p* < 0.0001	60.47 ± 17.36	*t* = 0.162 *p* = 0.851
	Bachelor's degree	92	33.92 ± 12.09	92.84 ± 5.63	*t *= 37.84 *p* < 0.0001	58.92 ± 14.93	
	Masters	3	27.77 ± 12.02	84.72 ± 2.4	*t* = 10.25 *p* = 0.009	56.94 ± 9.62	

Regarding other investigated variables (age, marital status, and education), statistically significant differences were observed before and 3 months after surgery within each group of patients. However, there were no statistically significant differences in the average percentage increase in ROE questionnaire scores between patients aged less than 25 years and those aged over 25 years before and after surgery (*p* = 0.079). Similarly, no significant differences were found in the comparison of average percentage increases in ROE questionnaire scores between single and married patients or patients with different educational level.

## 2 Discussion

This study aimed to evaluate patient satisfaction following rhinoplasty and to identify factors that influence it. A key finding, consistent with global research, was the high level of patient satisfaction with surgical outcomes. Analysis of ROE (Rhinoplasty Outcome Evaluation) questionnaire scores, collected before and 3 months after surgery, revealed a significant improvement in patient satisfaction regarding aesthetic results. The mean total ROE scores increased markedly postoperatively, with the difference being statistically significant (*p* < 0.001). These findings are in line with similar studies. For example, Bilgin et al. in Turkey assessed 60 patients undergoing primary rhinoplasty using the ROE questionnaire and reported an increase on average scores from 33.75 preoperatively to 87.9 postoperatively, a statistically significant improvement [7]. Similarly, studies by Esteves et al. and Haddady et al. also documented significant increases in ROE scores after rhinoplasty, reflecting high levels of patient satisfaction. Collectively, these findings suggest that rhinoplasty can lead to a substantial improvement in patient satisfaction, enhancing both nasal function and aesthetic appearance, thereby improving quality of life [4, 6].

This study involved 130 patients, mostly women under 25 years old, with a majority being university‐educated and single. These demographics align with previous findings; for example, Haddady et al. reported a predominance of well‐educated women with a mean age of 26.6 years among 60 Iranian patients [4]. Similarly, Loghmani et al. found over 70% women in two large cohorts undergoing elective rhinoplasty in Iran [11]. A study by Hama et al. in Iraq, a culturally similar country, also reported that over 90% of 65 patients were women, mostly aged between 18 and 49 years, with most having university education [12].

The high demand for rhinoplasty among young, single women can be attributed to social, cultural, and personal factors. Many societies emphasize female appearance and beauty standards, which pressures women to conform. In Iran, the dress code highlights the face, particularly the nose, making rhinoplasty especially popular among women compared to other cosmetic procedures [4]. This reflects broader trends of women prioritizing appearance and seeking cosmetic enhancements to meet cultural ideals.

This study found that the primary motivation for undergoing rhinoplasty among most patients (85.4%) was to improve the aesthetic appearance of the nose. Interestingly, neither gender nor level of education significantly influenced the reasons for surgery—whether cosmetic, functional, or both. However, age and marital status were found to significantly affect patient motivations. Younger individuals (under 25) mainly pursued rhinoplasty for cosmetic reasons, whereas older patients more frequently reported both aesthetic and functional (breathing‐related) concerns—a difference that was statistically significant (*p* = 0.002). Similarly, unmarried participants primarily sought cosmetic improvements, while married individuals were more likely to cite a combination of aesthetic and respiratory reasons, also showing statistical significance.

Comparative studies from other countries offer broader context. Esteves et al. in Portugal found that 82% of patients underwent rhinoplasty for both cosmetic and functional reasons, with only 5% motivated purely by aesthetics [6]. Likewise, Khan et al. in Pakistan reported that 51% of patients cited both cosmetic and breathing concerns, while 23% and 26% chose rhinoplasty for cosmetic and functional reasons, respectively [13]. Hama et al. in Iraq observed similar trends, with 55% motivated by aesthetics, 3% by breathing issues, and 42% by both [12]. These variations may reflect differing cultural values and healthcare priorities across regions.

This study found that the primary motivation for undergoing rhinoplasty among most patients (85.4%) was to improve the aesthetic appearance of the nose. Interestingly, neither gender nor level of education significantly influenced the reasons for surgery—whether cosmetic, functional, or both. However, age and marital status were found to significantly affect patient motivations. Younger individuals (under 25) mainly pursued rhinoplasty for cosmetic reasons, whereas older patients more frequently reported both aesthetic and functional (breathing‐related) concerns—a difference that was statistically significant (*p* = 0.002). Similarly, unmarried participants primarily sought cosmetic improvements, while married individuals were more likely to cite a combination of aesthetic and respiratory reasons, which was also statistically significant.

Comparative studies from other countries offer broader context. Esteves et al. in Portugal found that 82% of patients underwent rhinoplasty for both cosmetic and functional reasons, with only 5% motivated purely by aesthetics [6]. Likewise, Khan et al. in Pakistan reported that 51% of patients cited both cosmetic and breathing concerns, while 23% and 26% chose rhinoplasty for cosmetic and functional reasons, respectively [13]. Hama et al. in Iraq observed similar trends, with 55% motivated by aesthetics, 3% by breathing issues, and 42% by both [12]. These variations may reflect differing cultural values, societal expectations, and healthcare priorities across regions.

The findings of this study indicate that although patients with a diploma or lower educational qualifications showed a slightly greater increase in satisfaction scores, the difference was not statistically significant 3 months after rhinoplasty. This suggests that the level of education may not be a strong predictor of postoperative satisfaction in our sample.

Several studies have explored this relationship further. For example, Esteves et al. reported a significant difference in satisfaction based on education level, noting that patients with higher education levels expressed lower postoperative satisfaction scores [6]. Similarly, Ghorbani et al. found that a large proportion of dissatisfied patients were highly educated single women aged 30–34 [1]. Khan et al. also observed that patients with lower educational backgrounds reported significantly higher satisfaction following rhinoplasty (*p* < 0.01) [13].

Despite these findings, the evidence remains inconclusive. Satisfaction with cosmetic surgery is influenced by a range of factors—including expectations, personality traits, and social context—not solely education level. It is possible that individuals with lower education levels have fewer or more modest expectations, possibly due to limited access to medical information or online resources. However, generalizations should be avoided. Further research is warranted to better understand how education interacts with other variables to influence satisfaction outcomes in cosmetic surgery.

In this study, there was no statistically significant difference in ROE scores 3 months after rhinoplasty between patients under 25 and those over 25 years old (*p* = 0.079). This aligns with findings by Sismek et al., who also observed no significant age‐related differences in satisfaction after surgery [14].

However, other studies report contrasting results. Obeid et al. in Saudi Arabia found that patients over 35 were more satisfied with their outcomes [15]. Similarly, Balikci et al. in Turkey reported that while satisfaction did not differ significantly by age group, younger patients had lower pre‐ and postoperative satisfaction scores compared to older patients [16]. These mixed findings suggest that age alone may not predict satisfaction. Factors like expectations and psychological readiness may also play important roles.

This disparity in findings underscores the multifactorial nature of rhinoplasty, which involves both functional and aesthetic outcomes. Patient satisfaction can be influenced by a range of factors, including cultural expectations, psychological readiness, and individual perceptions. Age may contribute to satisfaction levels, as younger patients often have higher expectations shaped by societal beauty standards and self‐image concerns. Therefore, performing rhinoplasty in younger individuals requires careful patient selection and thorough preoperative counseling to align expectations with achievable results.

The primary objective of this study was to evaluate patient satisfaction following rhinoplasty and to identify factors influencing satisfaction levels. The results showed a generally high level of satisfaction 3 months after surgery, suggesting that, from the patient's perspective, the procedure is effective in achieving aesthetic and possibly functional goals. Furthermore, the significant associations found between certain variables and satisfaction levels highlight the importance of individualized surgical planning and patient selection. These findings contribute to the growing body of literature supporting the use of patient‐reported outcomes in assessing the success of cosmetic procedures and emphasize the relevance of incorporating patient expectations into preoperative evaluations.

This study has several limitations that should be considered when interpreting the results. First, it was conducted at a single center in Iran, which may limit the generalizability of the findings to other populations or healthcare settings. Second, although the sample size was adequate for statistical analysis, it may not fully represent the diversity of individuals undergoing rhinoplasty. Third, the follow‐up period was limited to 3 months postoperatively, which may not capture long‐term satisfaction or delayed complications. Additionally, all procedures were performed by a single surgeon, which helps reduce variability but may also limit the applicability of the findings to surgeries performed by practitioners with different levels of expertise or techniques.

Moreover, an increasing proportion of individuals seeking rhinoplasty today are from non‐Caucasian and African descent populations—groups that encompass diverse cultural, racial, and ethnic backgrounds. While the overarching goal in rhinoplasty is to achieve optimal aesthetic outcomes, it is essential to recognize and respect each group's unique anatomical characteristics and aesthetic ideals. For example, due to distinct nasal structures among patients of African descent, surgical planning and techniques should differ from those used in Caucasian patients to ensure higher satisfaction and more favorable outcomes [17].

Based on the findings of this study, several recommendations for future research are proposed. Future studies could examine morphological changes in the nose before and after surgery using image‐based analyses to better understand their impact on patient satisfaction. Investigating the effects of rhinoplasty on respiratory function and its influence on satisfaction levels would also be valuable. Additionally, comparing different rhinoplasty techniques, such as open versus closed approaches, may provide further insights into factors affecting patient outcomes.

This study focused solely on surgical rhinoplasty and did not include nonsurgical procedures; while comparisons with nonsurgical methods may offer additional understanding, they were beyond the scope of the current research and warrant future exploration. Furthermore, future research should consider how anatomical and aesthetic differences among ethnic groups—particularly between Caucasian and non‐Caucasian populations—affect satisfaction. Adopting a more individualized, ethnicity‐informed surgical approach could help achieve better outcomes and enhance the overall patient experience.

## 3 Conclusion

This study underscores that despite the intricate nature of rhinoplasty, it generally yields high satisfaction among patients. While certain demographic factors may influence patients' satisfaction levels with rhinoplasty outcomes, it can be asserted that rhinoplasty positively impacts the satisfaction of patients across various age groups, genders, and educational backgrounds. Given the significant role of the nose in facial aesthetics and considering cultural norms, particularly regarding women's attire in Iran, rhinoplasty is notably prevalent in Iran, particularly among women, compared to other cosmetic procedures.

The use of the ROE questionnaire emerges as a valuable tool for assessing patients' satisfaction post‐rhinoplasty, and its utilization is recommended for evaluating surgical outcomes. It is imperative to acknowledge that achieving complete satisfaction among all patients in cosmetic procedures is unrealistic. However, enhancing awareness of novel surgical techniques and attentiveness to patients' preferences can contribute to elevating satisfaction levels.

Moreover, the findings of this study are generally consistent with global research indicating high levels of patient satisfaction following rhinoplasty. For instance, studies conducted in various countries using the ROE questionnaire have also demonstrated favorable patient‐reported outcomes. However, certain cultural and social differences, such as the higher prevalence of rhinoplasty among Iranian women due to specific aesthetic expectations and societal norms, highlight the importance of context‐specific factors. Integrating these perspectives helps place our findings within the broader international discourse on rhinoplasty satisfaction.

Furthermore, by scrutinizing data and pinpointing areas of dissatisfaction with surgical results, surgeons and healthcare providers can enact changes and interventions to enhance the overall quality of rhinoplasty procedures. These measures may encompass refining surgical techniques, enhancing postoperative care protocols, and bolstering communication and support for patients throughout the process.

In conclusion, findings of this study can serve to optimize patient satisfaction, enhance the quality of rhinoplasty procedures, and equip surgeons with insights to deliver personalized and effective care to patients.

## Author Contributions

**Milad Rezai:** conceptualization, investigation, validation, visualization, software, data curation, methodology, writing – original draft. **Afrooz Haghdoost:** writing – original draft, methodology, validation, visualization, writing – review and editing, software, resources. **Mohammad Tolouei:** conceptualization, methodology, validation, writing – review and editing, project administration, supervision, data curation, resources, writing – original draft, funding acquisition. **Enayatollah Homaie Rad:** methodology, formal analysis, supervision, data curation, project administration, writing – original draft, resources. **Ramyar Farzan:** conceptualization, investigation, writing – original draft, writing – review and editing, methodology, validation, project administration, resources, supervision, funding acquisition. **Maria Ahmadi Jirandeh:** investigation, methodology, data curation, visualization, resources.

## Ethics Statement

All participants were fully informed of the study and Informed consent was obtained from all patients. The study was approved by the Ethics Committee of Research and Technology Deputy of Guilan University of Medical Sciences.

## Conflicts of Interest

The authors declare that the research was conducted in the absence of any commercial or financial relationships that could be construed as a potential conflict of interest. The funding sources and any potential conflicts of interest had no role in the study design, data collection, analysis, interpretation of data, writing of the report, or the decision to submit the manuscript for publication.

## Transparency Statement

The lead author Ramyar Farzan affirms that this manuscript is an honest, accurate, and transparent account of the study being reported; that no important aspects of the study have been omitted; and that any discrepancies from the study as planned (and, if relevant, registered) have been explained.

## Data Availability

The data that support the findings of this study are available from the corresponding author upon reasonable request. The data supporting this study's findings are available on request from the corresponding author.
